# Cluster analysis of men undergoing surgery for BPH/LUTS reveals prominent roles of both bladder outlet obstruction and diminished bladder contractility

**DOI:** 10.1371/journal.pone.0251721

**Published:** 2021-05-24

**Authors:** Andrew J. Schneider, Matthew Grimes, Will Lyon, Amanda Kemper, Sijian Wang, Wade Bushman

**Affiliations:** 1 Department of Urology, School of Medicine and Public Health, University of Wisconsin-Madison, Madison, Wisconsin, United States of America; 2 William S. Middleton Memorial Veterans Hospital, Madison, Wisconsin, United States of America; 3 Division of Geriatrics/Gerontology, Department of Medicine, Medical College of Wisconsin, Milwaukee, Wisconsin, United States of America; 4 Department of Mechanical Engineering, College of Engineering, University of Wisconsin-Madison, Madison, Wisconsin, United States of America; 5 Department of Statistics, Rutgers University, Piscataway, New Jersey, United States of America; Eberhard Karls Universitat Tubingen, GERMANY

## Abstract

Lower urinary tract symptoms (LUTS) in aging men are commonly attributed to bladder outlet obstruction from benign prostatic hyperplasia (BPH) but BPH/LUTS often reflects a confluence of many factors. We performed a hierarchical cluster analysis using four objective patient characteristics (age, HTN, DM, and BMI), and five pre-operative urodynamic variables (volume at first uninhibited detrusor contraction, number of uninhibited contractions, Bladder Outlet Obstruction Index (BOOI), Bladder Contractility Index (BCI) and Bladder Power at Qmax) to identify meaningful subgroups within a cohort of 94 men undergoing surgery for BPH/LUTS. Two meaningful subgroups (clusters) were identified. Significant differences between the two clusters included Prostate Volume (95 vs 53 cc; p-value = 0.001), BOOI (mean 70 vs 49; p-value = 0.001), BCI (mean 129 vs 83; p-value <0.001), Power (689 vs 236; p-value <0.001), Qmax (8.3 vs 4.9 cc/sec; p-value <0.001) and post-void residual (106 vs 250 cc; p-value = 0.001). One cluster is distinguished by larger prostate volume, greater outlet resistance and better bladder contractility. The other is distinguished by smaller prostate volume, lower outlet resistance and worse bladder contractility. Remarkably, the second cluster exhibited greater impairment of urine flow and bladder emptying. Surgery improved flow and emptying for patients in both clusters. These findings reveal important roles for both outlet obstruction and diminished detrusor function in development of diminished urine flow and impaired bladder emptying in patients with BPH/LUTS.

## Introduction

Aging men frequently develop lower urinary tract symptoms (LUTS). These symptoms are commonly attributed to bladder outlet obstruction by benign prostatic hyperplasia (BPH) and the clinical condition is termed BPH/LUTS. However, recent studies have implicated many factors in the pathogenesis of LUTS in addition to BPH. These include prostate inflammation and fibrosis, increased serum estrogen/testosterone ratio, diabetes, the metabolic syndrome, sleep apnea, polyuria and changes in bladder function. The confluence of these factors results in a broad spectrum of irritative and obstructive symptoms that are non-specific in terms of underlying etiology. Personalized medicine for BPH/LUTS requires first understanding the changes in lower urinary tract function that occur in men with BPH/LUTS. This can then be augmented by identifying the specific effects of superimposed medical and behavioral conditions.

We performed a cluster analysis of objective patient characteristics for men undergoing surgery for BPH/LUTS to identify meaningful subgroups within that surgical cohort. The analyzed parameters included patient age, Body Mass Index (BMI), history of diabetes mellitus or hypertension and pre-operative urodynamic metrics of bladder function and bladder outlet obstruction. This analysis identified two meaningful subgroups distinguished by robust differences in lower urinary tract anatomy and function that were associated with differences in objective surgical outcome measures, including resolution of retention, urinary flow rate and post-void residual urine volume.

## Results

### Patient cohort

**Fig 1:**
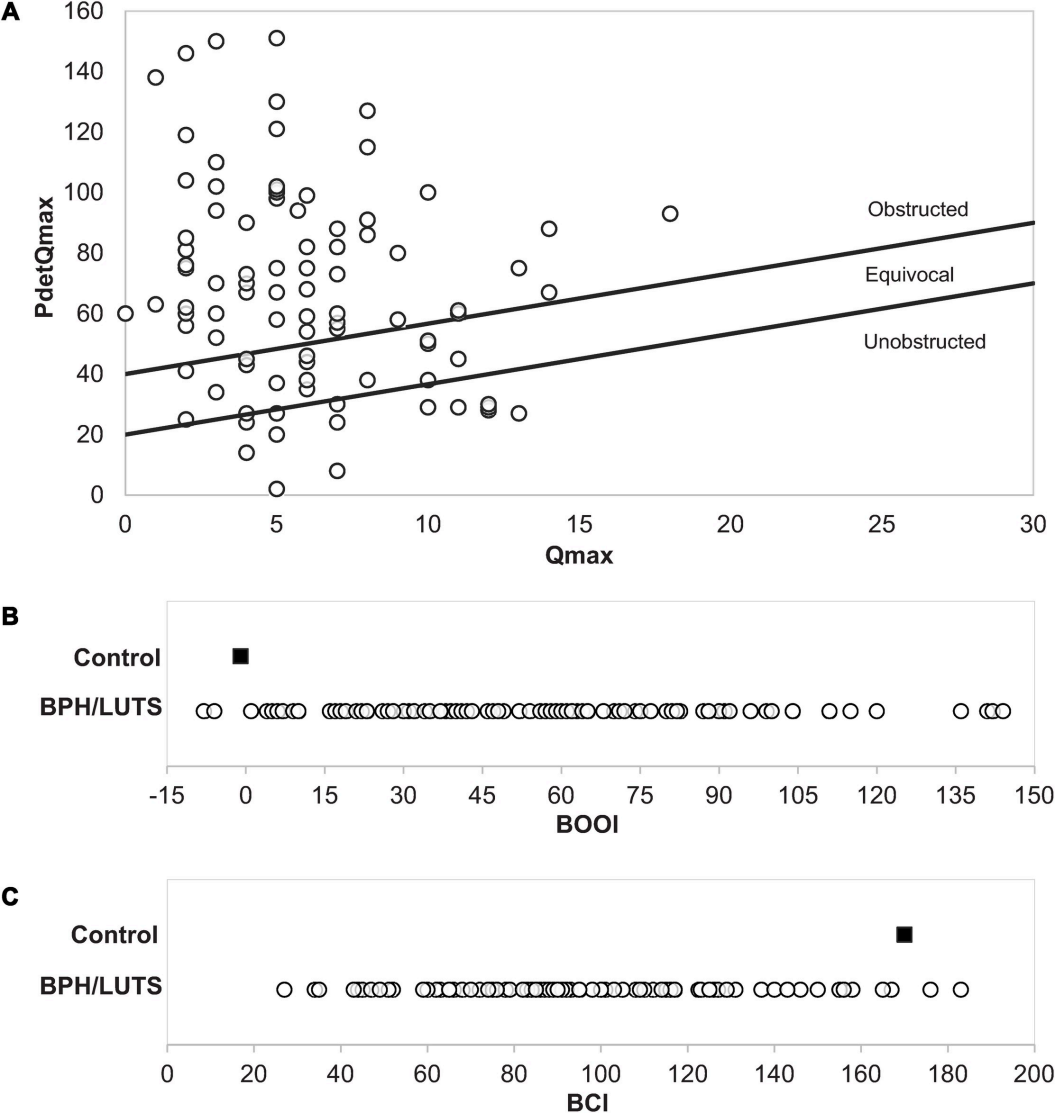
Preoperative urodynamic data. Open circle: BPH/LUTS patients. Filled Square are mean values calculated from published urodynamic data on young healthy controls (Schmidt et al., 2002). (A) ICS nomogram. (B) BOOI: Unobstructed < 20, Equivocal 20–40, Obstructed > 40. (C) BCI: Weak < 100, Normal 100–150, Strong > 150.

The patient cohort for this study was 94 men with BPH/LUTS who underwent surgery for having either failed medical therapy (n = 46) or experienced acute or chronic urinary retention requiring catheter drainage (n = 48). Patient data was collected under an IRB-approved protocol. Patients underwent either TURP (n = 80) or open simple prostatectomy (n = 14). Application of the ICS nomogram classified 63% of patients as obstructed, 20% as equivocal and 17% as unobstructed (Fig 1A). Urodynamic data were used to calculate the Bladder Outlet Obstruction Index (BOOI) and Bladder Contractility Index (BCI) for each patient that are plotted in Fig 1B and 1C. Nearly all patients exhibited increased BOOI as compared to normal young men and approximately two thirds of study patients exhibited significant bladder outlet obstruction (BOOI > 40) (Fig 1B). Nearly all patients exhibited decreased BCI as compared to normal young men and over half of the patients exhibited impaired bladder contractility (BCI < 100) (Fig 1C).

### Hierarchical cluster analysis

**Fig 2:**
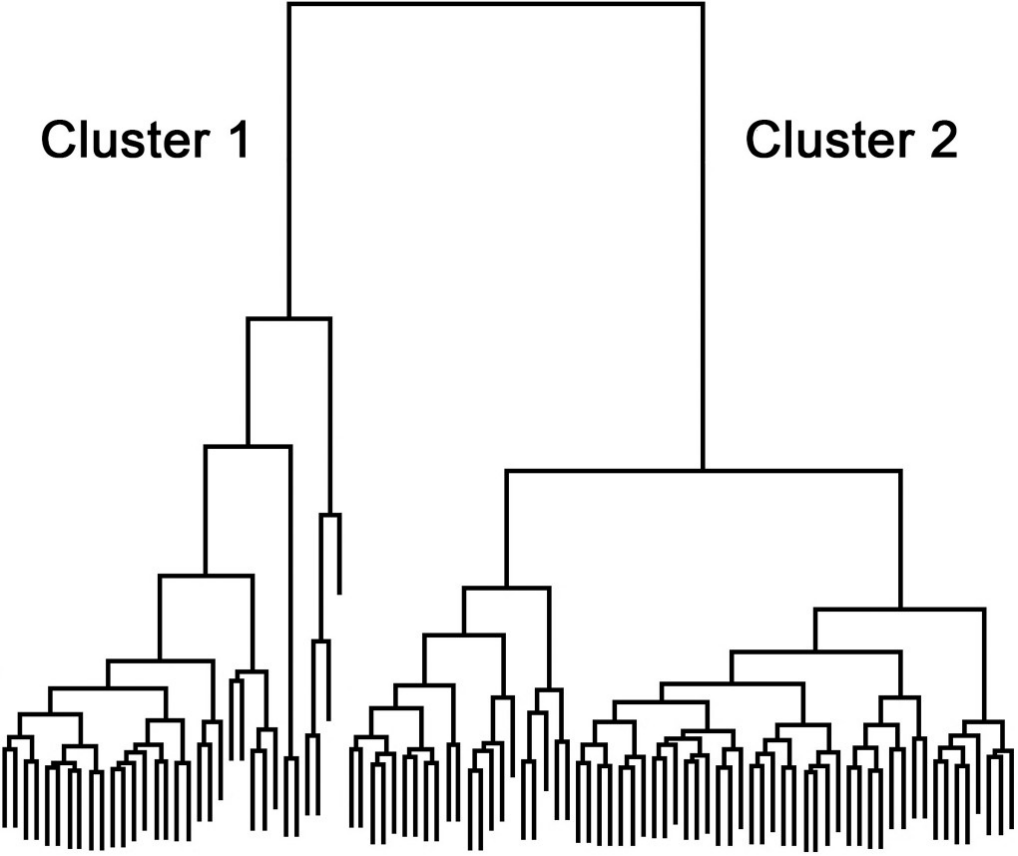
Hierarchical cluster analysis. Nine variables—age, HTN, DM, BMI, volume at first uninhibited detrusor contraction, number of uninhibited contractions during filling, BOOI, BCI, and bladder Power at maximum flow–were used for cluster analysis.

**Fig 3:**
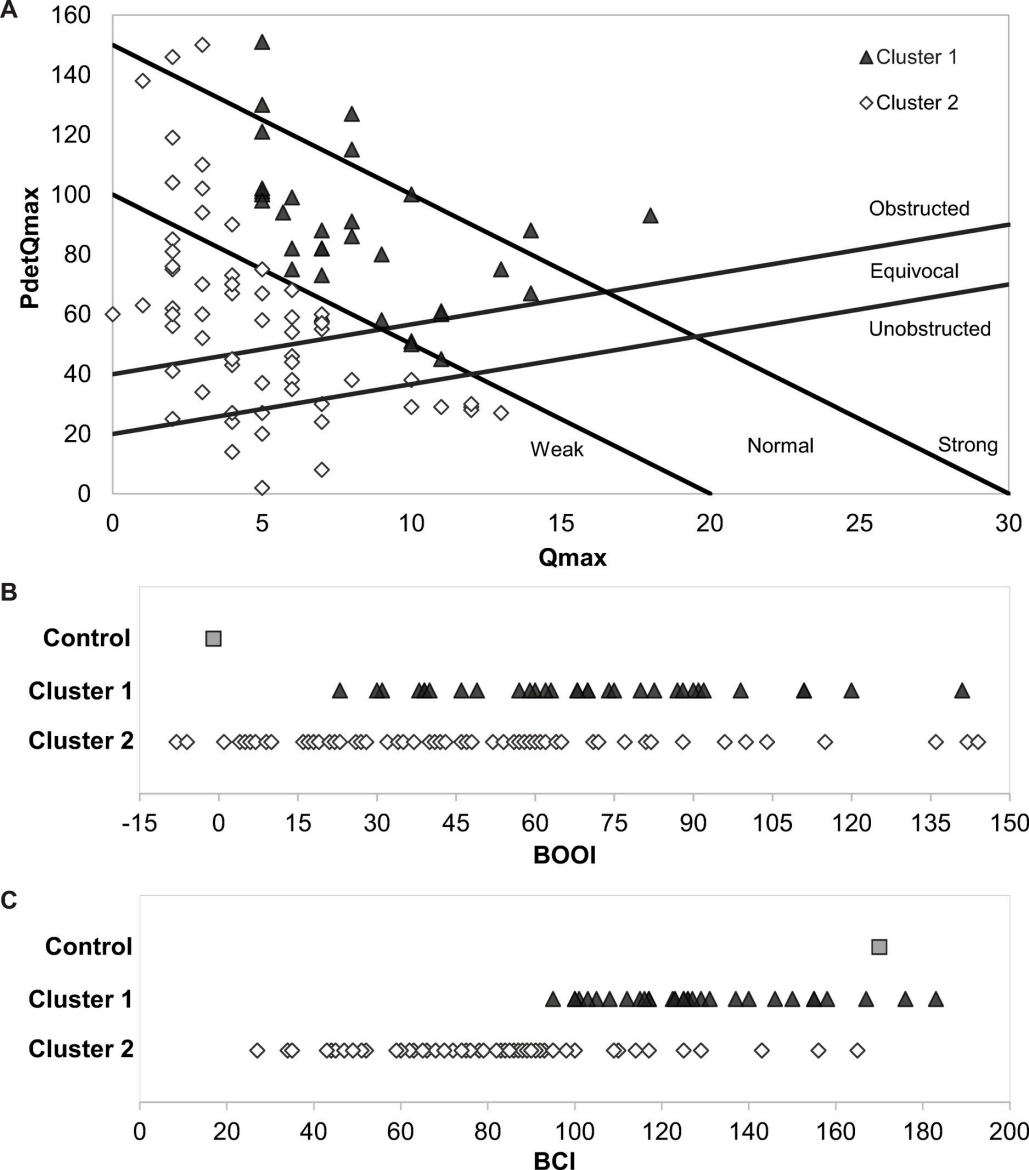
Preoperative urodynamic data sorted by cluster. Cluster 1: filled triangles. Cluster 2: open diamonds. Filled Square are mean values calculated from published urodynamic data on young healthy controls [1]. (A) ICS nomogram. (B) BOOI: Unobstructed < 20, Equivocal 20–40, Obstructed > 40. (C) BCI: Weak < 100, Normal 100–150, Strong > 150.

To determine whether discrete subpopulations were present in the patient cohort, we performed hierarchical cluster analysis on nine variables: age, HTN, DM, BMI, volume at first uninhibited detrusor contraction, number of uninhibited contractions during filling, BOOI, BCI, and bladder Power (S1 Dataset). This analysis identified two meaningful clusters in the patient cohort (Fig 2 and Table 1). Seven patients in each cluster underwent OSP; the remainder underwent TURP. Significant differences between Cluster 1 and Cluster 2 included mean Prostate Volume (95 cc vs 53 cc; p-value = 0.013), mean BOOI (70 vs 49; p-value = 0.002), mean BCI (129 vs 83; p-value < .0001) and mean Power (689 vs 236; p-value < .0001). Of note, PV was significantly correlated with BOOI (p = 0.03). Additional analysis revealed that the groups also differed significantly in opening pressure, Qmax, PdetQmax, voided volume, post-void residual (PVR) and bladder voiding efficiency (BVE). The mean number of uninhibited detrusor contractions were not significantly different between Cluster 1 and Cluster 2 (1.4 and 1.3, respectively). Among systemic factors included in the cluster analysis, diabetes mellitus was more prevalent in Cluster 2 (24% vs 3%; p = 0.01) as was hypertension but the difference was not significant (60% vs 44%, respectively). Patients in Cluster 2 were slightly older (66 vs 64 years) and mean BMI was the same in both (28). Comparison of BOOI in the two clusters revealed that 81% of men in Cluster 1 were obstructed compared to only 57% in Cluster 2 (p = 0.02; Fig 3B). More striking was the difference in bladder contractility. Whereas only one of 32 patients in Cluster 1 (3%) had diminished contractility, diminished contractility was present in 51 of 62 men in Cluster 2 (82%) (p<0.001; Fig 3C). In summary, our analysis reveals two discrete subgroups in the patient cohort. The smaller of the two subgroups is characterized by larger prostate volume, greater bladder outlet obstruction and more normal bladder contractility. The larger of the two subgroups is characterized primarily by diminished bladder contractility with smaller prostate volume and more modest bladder outlet obstruction. The distribution of the two clusters on the composite obstruction/contractility nomogram is shown in Fig 3A.

**Table 1:** Mean (and range) of urodynamic data and prostate volume for Clusters 1 and 2.

	Cluster 1 (n = 32)	Cluster 2 (n = 62)	p-value
BOOI	70.4 (23–141)	48.7 (-8–144)	0.001
BCI	129 (95–183)	82.7 (27–165)	< 0.001
Power	689 (420–1674)	236 (0–450)	< 0.001
Prostate Volume	94.5 (33–217)	52.9 (19–249)	0.001
Opening Pressure	85.7 (3–155)	58.5 (3–160)	< 0.001
Q_max_	8.3 (5–18)	4.9 (0–13)	< 0.001
P_det_Q_max_	87.0 (45–151)	58.4 (2–150)	< 0.001
Average Flow Rate	4.3 (2–13)	2.5 (1–7)	< 0.001
Voided Volume	274 (76–690)	163 (20–585)	< 0.001
PVR	106 (0–735)	250 (0–1360)	0.001
BVE	79.8 (40–100)	48.2 (3–100)	< 0.001

### Objective surgical outcomes

Of the 32 patients in Cluster 1, 12 (36%) had a history of retention and nine (28%) were catheter dependent at the time of surgery. Of the 62 patients in Cluster 2, 36 (58%) had a history of retention and 29 (47%) were catheter dependent at the time of surgery. The increased greater proportion of patients in Cluster 2 with history of retention and catheter-dependence at the time of surgery approached, but did not achieve statistical significance (p = 0.058 and 0.080, respectively). Only 3 patients remained catheter dependent post-operatively: one patient in Cluster 1 and two patients in Cluster 2. A total of 47 patients had free uroflowmetry performed pre- and post-operatively. Of these, 30 patients had a total bladder volume > 150 cc prior to voiding in both tests (14 and 16 in Clusters 1 and 2, respectively) and these data were used for analysis. These patients are representative both for the total patient population and each of the clusters as shown by comparison on the composite obstruction/contractility nomogram (S1 Fig). Surgery resulted in significantly increased Qmax, decreased PVR and improved BVE in both Clusters 1 and 2 (Table 2).

**Table 2:** Pre-operative and post-operative voiding metrics in representative subsets of patients in Clusters 1 and 2.

	Cluster 1			Cluster 2		
	(n = 14)			(n = 16)		
	Pre-Op	Post-Op	p-value	Pre-Op	Post-Op	p-value
	Mean ± SD	Mean ± SD		Mean ± SD	Mean ± SD	
Qmax	8.4 ± 3.2	21.2 ± 9.2	< 0.001	8.8 ± 5	15.3 ± 6	< 0.001
Voided volume	230 ± 161	266 ± 90.2	n.s.	246 ± 166	224 ± 119	n.s.
PVR	210 ± 296	57.9 ± 93.6	0.02	390 ± 387	180 ± 212	0.02
Bladder capacity	440 ± 304	324 ± 127	n.s.	636 ± 379	404 ± 267	0.01
BVE	63.1 ± 33.8	85.0 ± 18.2	0.04	45.5 ± 32.6	63.4 ± 22.9	0.03

Pre- and post-operative free uroflometry voiding metrics in patients whose pre-void bladder volumes were 150 ml or greater for both voids.

### Interaction between obstruction and contractility

**Fig 4:**
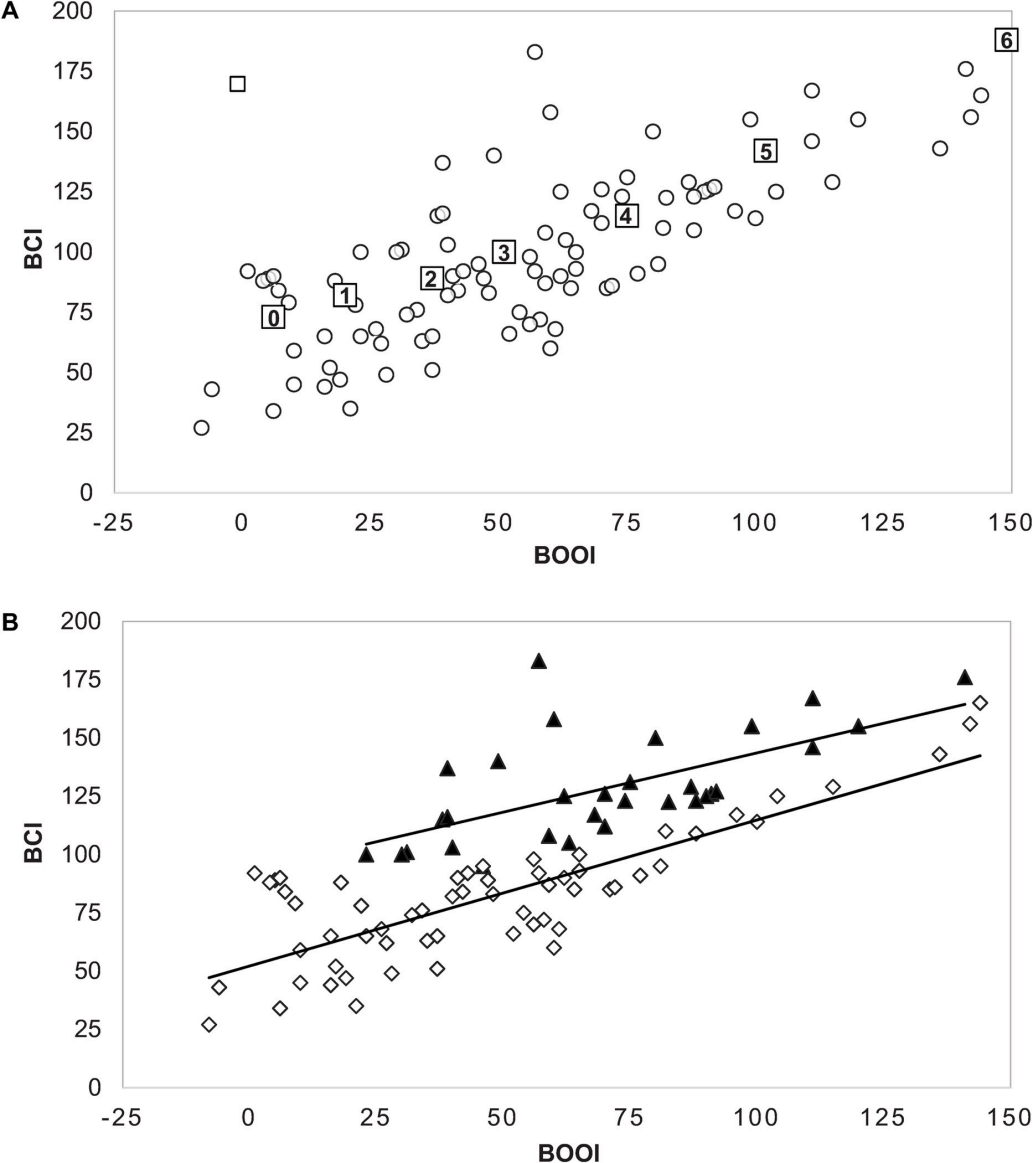
BOOI and BCI are positively correlated. (A) Plot of BCI vs BOOI for all 94 patients illustrating a positive correlation (r = 0.75). Data from Oelke et al. (2014) showing mean BCI and BOOI for Schaffer grades 1–6 in 786 men with LUTS suggestive of BPH is superimposed. (B) Plots of BCI vs BOOI for Cluster 1 (filled triangles) and Cluster 2 (open diamonds). Both clusters exhibit a positive correlation (r = 0.63, p< 0.001 and r = 0.82, p < 0.001, respectively).

To investigate the relationship of detrusor contractility to obstruction in our patient cohort, we plotted BCI as a function of BOOI for all 94 patients (Fig 4A). This revealed a clear-cut positive correlation. We then compared BCI as a function of BOOI among patients in Clusters 1 and 2 (Fig 4B). Both clusters exhibit a positive correlation and the slopes of the linear regressions for Cluster 1 and Cluster 2 (0.508 and 0.626, respectively) are not significantly different (p = 0.36).

## Discussion

Lower urinary tract symptoms in aging men have generally been considered the result of prostatic obstruction that induces irritative symptoms such as frequency, nocturia and urgency and produce obstructive symptoms such as hesitancy, diminished flow and incomplete bladder emptying. The primary rationale for bladder outlet surgery has been to remove prostate obstruction. However, not all men undergoing surgery are obstructed. In our cohort, 63% of patients were classified as obstructed, 20% as equivocal and 17% as unobstructed. This distribution is similar to other cohorts of patients selected for surgical treatment. For example, Min et al. [2] reported on urodynamic findings in 284 patients who underwent TURP: 57% were obstructed, 34% were equivocal and 9% were unobstructed. Another cardinal characteristic of this patient cohort was loss of contractility. Nearly all patients in our study exhibited diminished contractility as compared to young men and over half exhibited impaired contractility (BCI<100). These observations suggest operation of two factors in men with BPH/LUTS: increased outlet resistance and diminished detrusor contractility.

Our cluster analysis of objective patient characteristics identified two clusters. Obstruction was the dominant characteristic of Cluster 1; impaired contractility was rare. Diminished detrusor contractility was the distinguishing characteristic of patients in Cluster 2. Even so, pre-operative indices of urine flow and emptying were considerably worse for the patients in Cluster 2 than for patients in Cluster 1 (Table 1) and urinary retention was more common. These findings suggest that diminished detrusor contractility is also powerful determinant of flow and emptying. These same parameters improved post-operatively in both clusters, further suggesting that surgical decrease of outlet resistance provides benefit to patients where diminished contractility is a significant contributor to voiding dysfunction (Cluster 2).

What is the cause of diminished contractility in our patients? Loss of detrusor contractility from longstanding obstruction is intuitively appealing and supported by animal studies [3, 4]. However, there is a lack of published evidence that unrelieved bladder outlet obstruction in humans produces a profound loss of contractility. Indeed, Thomas et al. [5] followed 141 men with unrelieved bladder outlet obstruction for an average of 13.9 years and found a statistically significant but clinically insignificant decrease in contractility: mean BCI decreased from 134 to 126. Based on this, it seems unlikely that obstruction is primarily responsible for diminished contractility in our patient cohort, especially since over half of the men with impaired contractility (BCI<100) were unobstructed (BOOI <40; Fig 3A).

Aging as well as diabetes and the metabolic syndrome have been proposed as mechanisms for loss of detrusor contractility, but it is clear that aging plays a principal role. In a seminal contribution to the topic, van Mastrigt [6] reported on the association of aging with bladder contractility in both sexes. Bladder contractility decreased in a progressive and linear fashion with age in women. Men also exhibited an age-associated decrease in contractility but there was considerably more scatter in the data. This scatter was attributed by the author to the compensatory response of the bladder to outlet obstruction due to BPH. This interpretation has been echoed by subsequent authors. In our cohort there was a positive correlation between BCI and BOOI in the entire cohort, in both clusters and even among men with a BCI<100. This is not an artifact of patient selection for surgery since a similar trend was observed by Oelke et al. [7] in a large non-operative cohort of patients with BPH/LUTS. From these observations, we infer that the decreased contractility seen in most BPH/LUTS patients reflects normal bladder aging and that increased outlet resistance from BPH induces a compensatory detrusor hypertrophy that mitigates this loss of contractility.

The efficacy of surgery in patients with impaired contractility (BCI<100) has been debated. Thomas et al. [8, 9] observed no long-term symptomatic nor urodynamic benefits from TURP in patients with impaired contractility without obstruction. In contrast, Blaivas and colleagues [10] reported that surgery afforded significant improvement in symptoms and bladder emptying in men with impaired contractility. Good outcomes have also been reported by Tanaka et al. [11]. Cho et al. [12] reported that post-operative improvement in symptoms and flow in patients with impaired contractility were more likely to deteriorate over time. Taken together, these studies suggest that surgery is beneficial but less durable in these men. It is noteworthy then that impaired contractility with or without obstruction accounts for the majority of patients selected for surgical treatment in our practice. This may reflect a shift in patient profile due to an initial preference for medical therapy and minimally invasive procedures and begs the question if failure of medical therapy or minimally invasive procedures is more common in patients with impaired contractility than in patients with bladder outlet obstruction with good contractility.

**Fig 5:**
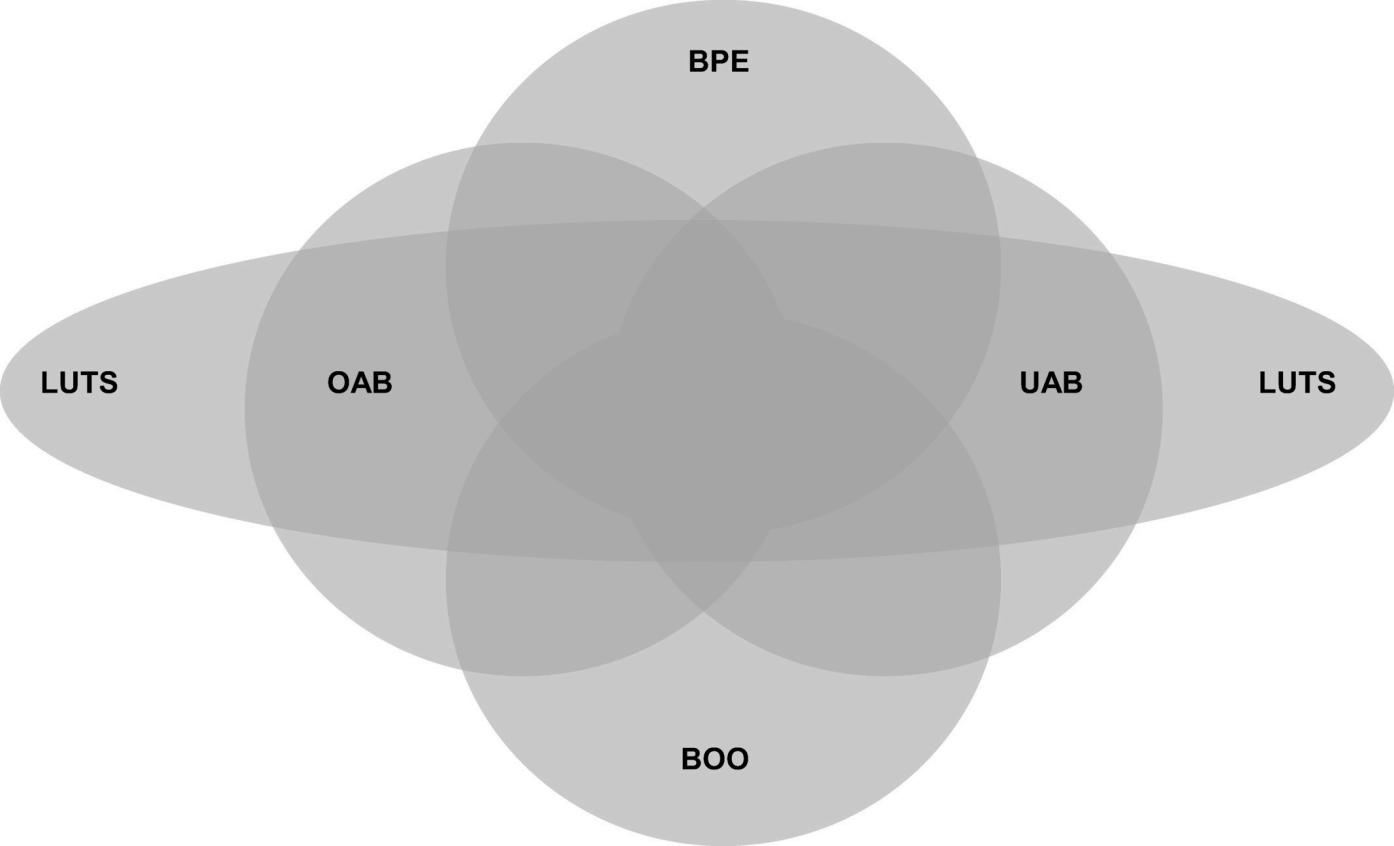
Venn diagram illustrating the complexity of BPH/LUTS. Symptoms variably overlap with benign prostate enlargement (BPE), bladder outlet obstruction (BOO), overactive bladder (OAB) and underactive bladder (UAB).

Loss of detrusor contractility is increasingly recognized as a major contributor to LUTS in aging men and may exist with or without concomitant bladder outlet obstruction (Fig 5). Our observations reveal diminished contractility as a major driver of the objective measures of both diminished flow and impaired emptying. This suggests and highlights the potential for novel bladder-specific ionotropic agents as a powerful addition to the medical treatment of men with BPH/LUTS. A benefit of surgery in patients with diminished contractility with or without outlet obstruction suggests a parallel to congestive heart failure. In that condition, where impaired cardiac muscle contractility is the major pathology, afterload reduction is the primary form of intervention and is beneficial even in the patients where the afterload is not abnormally increased [13, 14]. Surgery in men with diminished contractility may offer improvement by decreasing outlet resistance even when bladder outlet obstruction as strictly defined by urodynamic criteria is not present. Virtually all of our patients in our cohort exhibit decreased bladder contractility and increased outlet resistance as compared to normal controls (Fig 1). This suggests that development of LUTS is due to a combination of diminishing bladder contractility with age compounded by increasing outlet resistance. Surgery for BPH/LUTS has been traditionally termed as an intervention to correct “outlet obstruction”. However, considering confluent effects of diminished contractility and increased outlet resistance, it may be better considered a measure to reduce outlet resistance and decrease the bladder afterload.

Given the apparent positive effect of outlet obstruction on detrusor contractility, it is reasonable to consider the long-term impact of surgery on detrusor function in patients with impaired contractility. Our limited follow-up does not allow us to address this question and we are not aware of any published study that directly addresses this question. However, a study of long-term outcomes of TURP in men with outlet obstruction and normal contractility may be pertinent. In that study, Thomas et al. [9] found that over a third of men developed impaired contractility and that this was highly correlated with symptomatic failure. Notably, predictive factors for development of impaired contractility were decreased detrusor contractility and lesser degree of obstruction. These observations stand in apparent contrast to the relative stability of symptoms and bladder contractility in a companion study of long-term outcomes in men with obstruction who did not undergo surgery [15]. Taken together, these suggest that outlet resistance may work to maintain detrusor contractility and that relief of obstruction in patients with relatively diminished contractility may actually be detrimental to detrusor function in the long-term.

Our study highlights the prominent role of impaired contractility among patients in a modern surgical cohort and make clear the need to consider contractility as well as obstruction in the pre-surgical evaluation of men with BPH/LUTS. Further, our analysis of the interaction between obstruction and contractility should draw attention to the potential implications of that dynamic interplay for treatment outcomes particularly in patients with impaired contractility. We hope that future studies comparing urodynamic findings as well as symptoms pre-operatively and at long-term follow-up will illuminate this issue.

A weakness of this retrospective study is that it includes patients electing surgical intervention as a product of shared decision making between patients and five different surgeons. The decision for surgery was therefore not according to any standard criteria. Mitigating this is that nearly half the patients have a history of urinary retention and therefore a strong indication for surgery and this subgroup exhibited the same attributes as the patients without history of retention. Reporting of symptom scores and symptom improvement with surgical treatment is a standard feature of BPH/LUTS studies and have been reported numerous times for surgical treatment of similar patient cohorts. These are important metrics for judging the success of surgery, however, they are subjective and not specific for any etiologic mechanism. The primary focus of this study was to identify meaningful subgroups within a surgical cohort defined by objective criteria. Secondary points of analysis included the relationship between contractility and obstruction in the subgroups and correlation with preoperative and postoperative indices of urine flow and emptying. Future analysis will correlate symptoms and symptomatic outcomes with the two clusters and with the successive nodes in the dendrogram.

## Methods

Through a non-selective review of billing information obtained through the EPIC medical information system at the University of Wisconsin Hospital and Clinics (requested and received in 2016), we identified men who underwent surgery for BPH/LUTS between 2005 and 2016. The inclusion criteria were preoperative urodynamic evaluation and the availability of complete pre-and post-operative clinical information. Exclusion criteria were inability to demonstrate a significant voluntary detrusor contraction on urodynamic evaluation or evidence of prostate cancer involving greater than 5% of the resected tissue. A total of 94 patients were identified satisfying these criteria. Urodynamic evaluation was performed by one physician (WB) in accordance with the standards of the ICS but no specific recommendations regarding surgical management was made. Surgery was performed by five different surgeons and the decision for surgery was the product of shared decision-making in which the urodynamic data was only one factor considered in counseling the patient. Study data was collected under an IRB-approved protocol and under a waiver of informed consent that was granted by the University of Wisconsin Health Sciences IRB because the information was originally collected for standard of care purposes. All methods including urodynamic and clinical evaluation, imaging studies and histologic evaluation were performed in accordance with the relevant guidelines and regulations. Prostate volume, calculated from CT using the ellipsoid formula, was available for 56 men. Comprehensive clinical and urodynamic data was abstracted from the medical record.

All urodynamic evaluations were performed with Medical Microsystems (MMS). Each investigation consisted of spontaneous free-flow uroflometry, medium-fill (50 cc/min) cystometry performed with room temperature fluid through a 7 French catheter and subsequent pressure-flow study in the standing position. Abdominal pressure was recorded through a 9 French rectal catheter. Sphincter electromyographic activity was recorded from cutaneous electrodes placed on the perineum to monitor for dysfunctional sphincter activity during voiding (none was observed). Both bladder and rectal catheters were connected to microtip transducers zeroed to the level of the symphysis pubis. Typically, two or more pressure-flow studies were performed. All the data used for analysis was from the first study performed unless precluded by artifact, in which case the data was obtained from the first subsequent study free of artifact.

The following metrics were determined from the urodynamic data: maximum flow rate (Qmax), detrusor pressure at maximum flow (PdetQmax), Bladder Outlet Obstruction Index (BOOI) was calculated from the formula BOOI = PdetQmax– 2Qmax, Bladder Contractility Index (BCI) was calculated from the formula BCI = PdetQmax + 5Qmax, Bladder Power at maximum flow was calculated from the formula Power = (PdetQmax)(Qmax) [16] and Bladder Voiding Efficiency (BVE) was calculated from the formula BVE = Voided volume divided by voided volume plus post-void residual (PVR). For comparison purposes we used previously published urodynamic data from young men with an average age of 25 years as controls [1]. All data is available in S1 Dataset.

Statistical Methods: Hierarchical clustering with complete linkage was conducted on the 94 patients. First, the Euclidean-distance based on 9 variables (age, HTN, DM, BMI, volume at first uninhibited detrusor contraction, number of uninhibited contractions during filling, BOOI, BCI, Bladder Power) was calculated to define the dissimilarity between each pair of patients. The hierarchical clustering procedure starts by assigning each patient to a cluster. Then it finds the closest (most similar) pair of clusters and merges them into a single cluster. With the usage of the complete-linkage, the distance between one cluster and another cluster is considered to be the greatest distance from any patient of one cluster to any patient of the other cluster. The procedure continues until all patients are merged into a single cluster. Fig 2 shows the dendrogram, a cluster tree built by hierarchical clustering to represent all 94 patients. On the dendrogram, each “node” represents a group of patients, which links to two or more successor nodes (groups of patients).

To identify correlations, Pearson’s Correlation Coefficient (r) and its significance (p) were calculated. To determine if the proportion of patients a) with obstruction or impaired contractility, or b) with/without diabetes mellitus was significantly different between Cluster 1 and Cluster 2, Chi-squared test was used to calculate p-value. To determine if means of UDS measurements and prostate volumes were significantly different between Cluster 1 and Cluster 2 patients, Mann-Whitney U-test was used to calculate p-value. To determine if mean free uroflometry measurements pre- and post-operatively were significantly different, Wilcoxon Signed Rank test was used to calculate p-value. To determine if the regression slopes for Cluster 1 and Cluster 2 on the BCI vs BOOI scatterplot were significantly different, the Regression Slope Test was used to calculate the p-value. P ≤ 0.05 was considered significant.

## Supporting information

S1 Fig Distribution of Cluster 1 and Cluster 2 patients who were included in the comparison of post-operative metrics presented in Table 2. ICS nomogram–PdetQmax vs Qmax–with data from all 94 patients. Cluster 1 patients (black triangles) and Cluster 2 patients (open diamonds) with free uroflometry performed pre- and post-operatively. Patients with gray symbols were not included in comparison of post-operative metrics.
(TIF)

S1 Dataset Data collected from medical records of a cohort of 94 men undergoing surgery for BPH/LUTS. The first tab contains only the 9 variables data used for cluster analysis. The second tab contains all data presented in this manuscript.
(XLSX)
