# Supplementary figures and images for: Cluster analysis of men undergoing surgery for BPH/LUTS reveals prominent roles of both bladder outlet obstruction and diminished bladder contractility
Source: PLoS One. 2021 May 24;16(5):e0251721. doi: 10.1371/journal.pone.0251721 (PMC8143400; doi:10.1371/journal.pone.0251721)

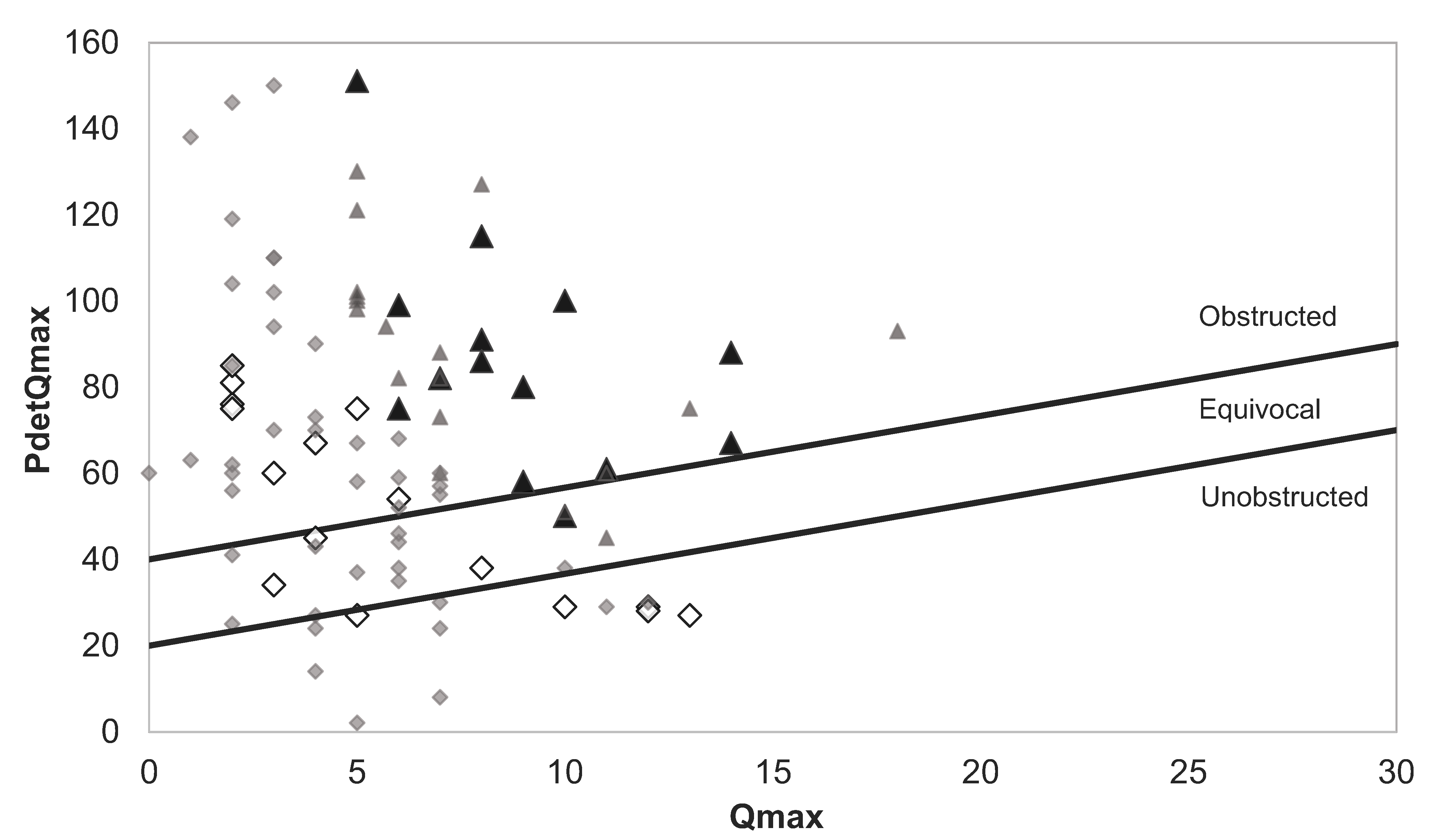

Supplement: S1 Fig — ICS nomogram–PdetQmax vs Qmax–with data from all 94 patients. Cluster 1 patients (black triangles) and Cluster 2 patients (open diamonds) with free uroflometry performed pre- and post-operatively. Patients with gray symbols were not included in comparison of post-operative metrics. (TIF) [file pone.0251721.s001.tif]
